# SDA, a DNA Aptamer Inhibiting E- and P-Selectin Mediated Adhesion of Cancer and Leukemia Cells, the First and Pivotal Step in Transendothelial Migration during Metastasis Formation

**DOI:** 10.1371/journal.pone.0093173

**Published:** 2014-04-03

**Authors:** Rassa Faryammanesh, Tobias Lange, Eileen Magbanua, Sina Haas, Cindy Meyer, Daniel Wicklein, Udo Schumacher, Ulrich Hahn

**Affiliations:** 1 Hamburg University, MIN-Faculty, Chemistry Department, Institute for Biochemistry and Molecular Biology, Hamburg, Germany; 2 University Medical Center Hamburg-Eppendorf, University Cancer Center, Institute of Anatomy and Experimental Morphology, Hamburg, Germany; Wayne State University School of Medicine, United States of America

## Abstract

Endothelial (E-) and platelet (P-) selectin mediated adhesion of tumor cells to vascular endothelium is a pivotal step of hematogenous metastasis formation. Recent studies have demonstrated that selectin deficiency significantly reduces metastasis formation *in vivo*. We selected an E- and P-*S*electin specific *D*NA *A*ptamer (SDA) via SELEX (*S*ystematic *E*volution of *L*igands by *EX*ponential enrichment) with a *K*_d_ value of approximately 100 nM and the capability of inhibiting the interaction between selectin and its ligands. Employing human colorectal cancer (HT29) and leukemia (EOL-1) cell lines we could demonstrate an anti-adhesive effect for SDA *in vitro*. Under physiological shear stress conditions in a laminar flow adhesion assay, SDA inhibited dynamic tumor cell adhesion to immobilized E- or P-selectin. The stability of SDA for more than two hours allowed its application in cell-cell adhesion assays in cell culture medium. When adhesion of HT29 cells to TNFα-stimulated E-selectin presenting human pulmonary microvascular endothelial cells was analyzed, inhibition via SDA could be demonstrated as well. In conclusion, SDA is a potential new therapeutic agent that antagonizes selectin-mediated adhesion during metastasis formation in human malignancies.

## Introduction

Despite all advances in molecular medicine, the treatment of metastatic disease is still an unresolved problem, thus the mortality of cancer is still high. In 2013, about 1,600,000 incidences of cancer and 580,000 cancer deaths were registered alone in the United States [1]. This high mortality rate is mostly due to the fact that metastasized cancer cannot be treated curatively. Thus, more than 90% of the cancer victims die because of metastatic spread of the malignant cells [2]. Hence, the advancement in cancer therapy essentially depends on the therapeutic manipulation of the metastatic process.

**Figure 1:**
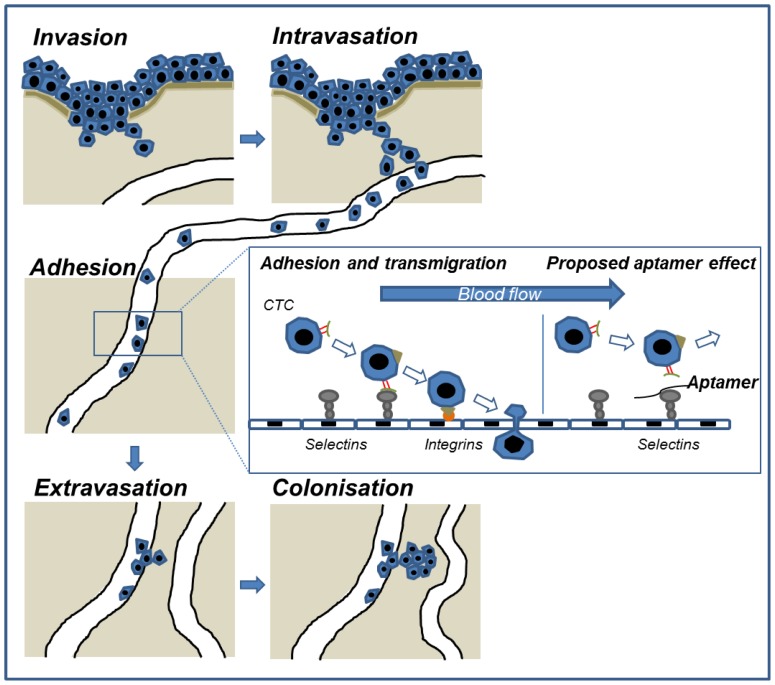
Adhesion of circulating tumor cells towards vascular endothelium is a critical step in metastasis formation. Metastatic spread of cancers occurs via a sequential process that begins with the invasion of primary tumor cells. After crossing the basement membrane and migrating through the adjacent connective tissue, certain tumor cells intravasate into tumor microvessels and circulate with the blood stream to distant organs. At the future metastatic site, these circulating tumor cells (CTC) are slowed down from the blood stream and adhere to vascular endothelium. This step is crucially initiated by interactions between endothelial selectins and certain carbohydrate ligands such as sialylated Lewis structures presented at the tumor cell surface. Adhesion is a prerequisite for extravasation and subsequent colonisation of the metastatic tissue. Selectin-binding aptamers that impair the interaction between selectin ligands and endothelial selectins would be therefore a promising new anti-metastatic therapeutic.

During haematogeneous metastasis formation tumor cells have to adhere to and transmigrate the endothelium at the target site of the future metastasis. In doing so, they mimic the behavior of normal leukocytes as they use the selectin-mediated migratory pathway of leucocytes in inflammation [3], [4]. The calcium dependent selectins are composed of a lectin domain, which is responsible for the ligand binding, an epidermal growth factor-like domain and a flexible number of consensus repeat units [5], [6]. Cancer cells adhere on the endothelium by interacting with endothelial (E-) and platelet (P-) selectins [3], [4] following transmigration into the underlying tissue and subsequent proliferation (Figure 1). In a xenograft model of human pancreatic adenocarcinoma, the absence of E- and P-selectins led to 85% reduction of peritoneal carcinomatosis [7]. A similar reduction of metastasis was observed in an eosinophilic and chronic myelogeneous xenograft model [8] and a colorectal carcinoma model in the absence of both selectins [4]. Thus, blocking the selectin-mediated adhesion mechanism in hematogenous as well as in intraperitoneal metastasis is of clinical significance and may be a potential therapeutic approach against pathophysiological events (Figure 1). So far, monoclonal antibodies, glycomimetic antagonists [9], and different modified aptamers [10]–[12] have been tested as selectin inhibitors in pre-clinical trials.

Aptamers are DNA or RNA oligonucleotides with defined three-dimensional structures displaying high affinity and specificity for target molecules. With respect to their binding characteristics, they are comparable to antibodies. The advantages of aptamers over antibodies are (i) their simple and affordable synthesis, (ii) the possibility for their long-term storage at room temperature and thus (iii) their stability during shipping, (iv) the diverse possibilities of conjugating to other reagents and (v) their lack of immunogenicity [12]. Aptamers are selected in an *in vitro* process called *S*ystematic *E*volution of *L*igands by *EX*ponential Enrichment (*SELEX*) by amplifying DNA or RNA oligonucleotides based on their affinity for a target molecule [13]–[15]. The diversity of oligonucleotides allows the selection of aptamers for almost any target molecule. Thus, aptamers are a suitable tool for medical use in diagnosis and therapy.

We started to select DNA aptamers aiming at their medical application in inhibiting tumor metastasis. We succeeded in selecting an aptamer with high affinity for E- and P-selectin and tested the binding to its target molecules via filter retention assays (FRA). To assess the aptamer's ability to block the interaction of cancer and leukemia cells with selectins, we used laminar flow assays under physiological shear stress conditions, employing human colorectal cancer cell line HT29, human chronic eosinophilic leukemia cell line EOL-1, and primary human pulmonary microvascular endothelial cells (HPMECs). The selected DNA aptamers inhibited the interaction of cancer cells with the endothelium by blocking their selectin-mediated adhesion. Thus the selected aptamers provide an attractive alternative to existing comparable reagents.

## Materials and Methods

### Oligonucleotides

The initial DNA library used for SELEX was purchased from Metabion. It consisted of a randomized region of 50 nucleotides flanked by constant regions at the 5′- as well as 3′-end (21 or 20 nucleotides, respectively) to allow for PCR amplification. Further oligonucleotides were purchased from Invitrogen.

### Biotinylation of E-selectin and Immobilization on Streptavidin-coated Dynabeads (Target Beads)

For biotinylation reaction, 50 μg recombinant human E-selectin/IgG-Fc-chimeras (rh E-selectin; R&D Systems) were incubated with threefold molar excess of sulfo-NHS-LC-biotin (Thermo Scientific) as described in detail in the manufactureŕs manual. Finally, the biotinylated rh E-selectin was immobilized on 5 mg streptavidin-coated magnetic beads (Dynabeads; life technologies) and suspended in selection buffer (3 mM MgCl_2_ in phosphate buffered saline (PBS), pH 7.5) including 1 mg bovine serum albumin (BSA)/mL as previously described [16].

### Biotin Immobilization on Streptavidin-coated Dynabeads (Pre-selection Beads)

Streptavidin-coated magnetic beads (5 mg) were washed five times with 500 μL washing buffer (1 mg BSA/mL PBS), resuspended in 500 μL PBS containing 0.9 mM biotin, and incubated for 30 minutes at room temperature. The biotin-streptavidin-coated magnetic beads were washed five times with 500 μL washing buffer, resuspended in 1.5 mL PBS including 1.25 mg BSA/mL and were subsequently used for pre-selection.

### *In vitro* Selection of Selectin Binding DNA Aptamers

**Figure 2:**
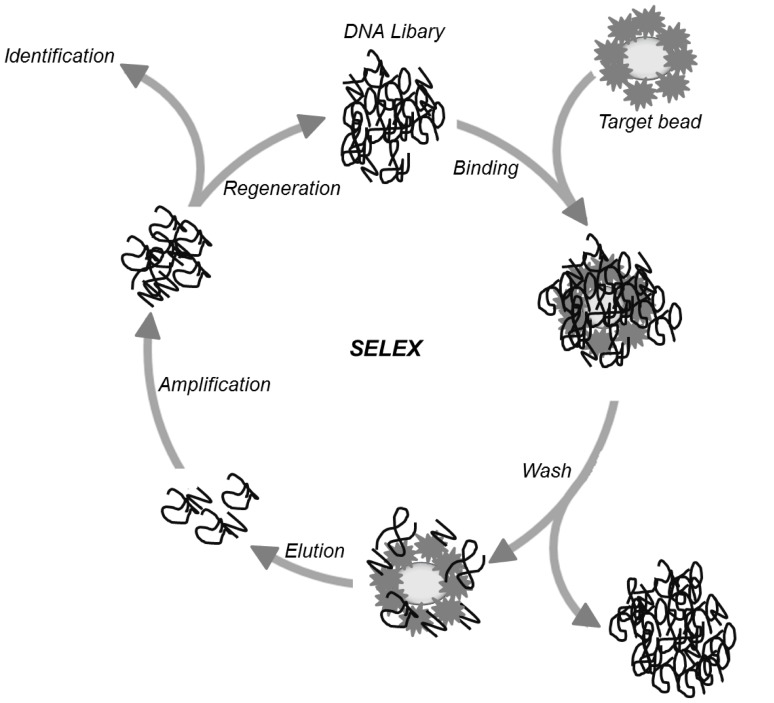
Systematic Evolution of Ligands by Exponential Enrichment (SELEX). DNA was incubated with immobilized rh E-selectin on magnetic beads (target beads). After washing and removing unbound DNA, target bound DNA was eluted and amplified via PCR. The double-stranded PCR product was separated in single strand DNA and the next SELEX step started after this regeneration. The identification of aptamers via cloning and sequencing of the enriched ssDNA pool was performed after several SELEX rounds (10–20 rounds).

For *in vitro* selection of E-selectin specific DNA aptamers, we used the SELEX procedure (Figure 2) including a pre-selection step to remove streptavidin-binding DNA. For pre-selection, 250 pmol of the ssDNA library (5′-GCCTGTTGTGAGCCTCCTAAC-(N)_50_-CATGCTTATTCTTGTCTCCC-3′) were incubated with 30 nmol pre-selection beads for 30 minutes at room temperature. Streptavidin-binding DNA was removed via magnetic separation. The first round of selection was initiated by incubation of pre-selected DNA library with 50 pmol target beads for 30 minutes at room temperature. After removing of unbound DNA by washing with 200 μL selection buffer, bound DNA was eluted in 55 μL water by heating the mixture to 80°C for 3 minutes. Eluted DNA was amplified via PCR using 1 μM 5′-biotinylated reverse primer (5′-GGGAGACAAGAATAAGCATG-3′), 1 μM forward primer (5′-GCCTGTTGTGfAGCCTCCTAAC-3′), 1.5 mM MgCl_2_, 200 μM dNTPs in 1×PCR buffer B and 0.05 U FIREPol DNA Polymerase per μL (the latter two purchased from Solis BioDyne). Separation of single-stranded DNA aptamers from double-stranded PCR products was done by streptavidin-coated magnetic beads. Therefore, PCR products were diluted 1∶2 in 2×B&W buffer (1 mM EDTA, 2 M NaCl in 10 mM Tris-HCl, pH 7.5), added to streptavidin-coated magnetic beads and subsequently washed twice with 1×B&W buffer. After incubation for 15 min at RT the supernatants were removed and beads were re-suspended in 150 mM NaOH following incubation for another 10 minutes at room temperature. The combined supernatants, containing the single-stranded DNA, were neutralized using 100 mM HCl and utilized as start pool for a next selection round. After incubation of DNA single strands with target beads the amounts of washing steps were raised from round to round increasing stringency.

### Cloning

Isolation of selected aptamers was accomplished by molecular T-A-cloning. Therefore, the vector pUC19-T (pCR2.1-TOPO; Invitrogen) was restricted with XcmI (Thermo Scientific) to generate 3′ thymidine overhangs [17], [18]. Aptamers were amplified via PCR using FIREPol DNA Polymerase producing 5′ adenosine overhangs and ligated with the vector using T4 DNA ligase (Thermo Scientific). DNA of 50 resulting clones was isolated and sequenced (GATC-Biotech).

### Filter Retention Assay

Filter retention assays (FRA) were used to determine dissociation constants of the aptamer selectin interactions. Therefore, DNA was radioactively labeled with [<$>\raster="rg1"<$>-^32^P] adenosine-5′-triphosphte (Hartmann Analytic) using T4 polynucleotide kinase (Thermo Scientific) and purified via gel extraction followed by isopropanol precipitation. For binding assays, constant amounts of radioactive DNA (<1 nM) was incubated with increasing amounts of corresponding proteins (0–1 μM) for 30 minutes at room temperature in selection buffer. Afterwards protein-aptamer complexes were filtrated (Manifold I Dot-BlotSystem, Whatman) through a pre-equilibrated (15 minutes in 0.4 M KOH) nitrocellulose membrane (Whatman) for 5 minutes in demineralized water at room temperature and then washed in selection buffer. After filtration, the nitrocellulose membrane was dried and exposed for 3 hours to a phosphor imaging screen (Bio-Rad). For quantification we used the One site-Specific binding model (Quantity One software).

### Nuclease-resistance-stability

DNA aptamer was radioactively labeled with [?-^32^P] adenosine-5′-triphosphte as described above and incubated with 100 μL full medium at 37°C. After definite time points (0–720 min), 10 μL of each sample were frozen in liquid nitrogen and subsequently analyzed via 10% denaturing gel electrophoresis.

### Cell Lines and Culture Conditions

The human colorectal cancer cell line HT29 (purchased from the European Cell Culture Collection) and the human chronic eosinophilic leukemia cell line EOL-1 (from DSMZ) were maintained in RPMI-1640 supplemented with 2 mM L-glutamine, 10% fetal calf serum (FCS), 100 μg penicillin/mL and 100 μg streptomycin/mL (latter reagents were purchased from PAA) at 37°C in a humidified atmosphere of 5% CO_2_. For all assays, HT29 cells were cultivated until 80% confluency; EOL-1 cells were grown in suspension. Primary human pulmonary microvascular endothelial cells (HPMECs) were obtained from Promocell, cultured in endothelial cell growth medium MV supplemented with the corresponding supplement mix as provided by the supplier, and 100 μg penicillin/mL and 100 μg streptomycin/mL. HPMECs were sub-cultured using a specific detach kit (PromoCell) according to the manufacturer’s instructions. All experiments with primary cells were performed during the first six passages.

### Inhibition of Dynamic Tumor Cell Adhesion to Immobilized Human E- and P-selectin

We analyzed the aptamer-mediated inhibition of selectin ligand interaction under laminar flow conditions representing endothelial shear stress found in post-capillary venules of metastatic organs such as lungs [19]. For this purpose, rh E- and rh P-selectin were immobilized on IBIDI treat microslides VI (IBIDI) at a final concentration of 0.02 mg/mL, diluted in DPBS including Ca^2+^ and Mg^2+^ (DPBS^+^; PAA) for 30 min at 37°C. In parallel as a control, chambers were coated likewise with 0.02 mg/mL IgG-Fc (R&D Systems). Slides were washed with 50 μL DPBS and incubated with or without aptamer (0.15 mg aptamer/mL DPBS^+^) for 30 minutes at room temperature. IBIDI microslides capillaries were washed again with 50 μL DPBS^+^ and then perfused with 1×10^5^ HT29 tumor cells per mL at a laminar flow rate of 8.5 mL/h [19]. Adhesive events were recorded and subsequently analyzed as described before [4], [20], [21].

### Inhibition of Dynamic Tumor Cell Adhesion to Human Pulmonary Endothelium

To determine the inhibitory potential of our aptamer on shear-resistant tumor cell adhesion towards human pulmonary endothelium, IBIDI treat microslides VI were coated with confluent HPMEC monolayers, which remained untreated or were stimulated with recombinant human (rh) TNFα (Peprotech) for 4 h prior to the flow adhesion assay (10 ng/mL). The stimulated HPMECs were incubated with 0.15 mg aptamer/mL medium for 30 minutes at 37°C and then perfused with 1×10^5^ HT29 tumor cells per mL as described above (control without aptamer).

### Statistics

For all statistical analyses Graphpad Prism 6 (La Jolla California, USA) was used. The values are given as mean ± standard deviation. The One site-Specific binding model (Graphpad Prism 6) was used for calculating the dissociation constants. The two-tailed unpaired t-test was used for comparisons between two groups. Significant differences between two means with p<0.05 are marked with *, very significant differences (p<0.01) with ** and extremely significant differences (p<0.001) with ***.

## Results

### Selection and Characterization of an E- and P-selectin Specific DNA Aptamer

**Figure 3:**
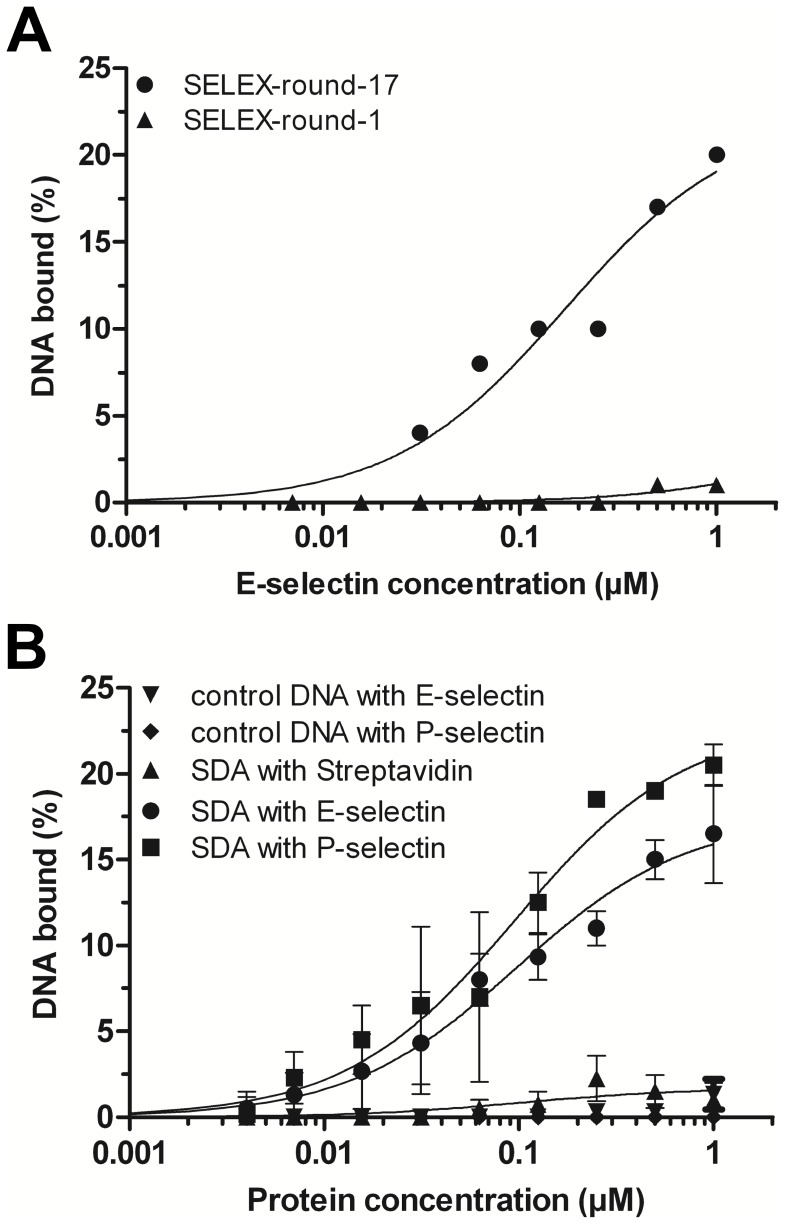
Affinities of selected DNA aptamers to rh E- and rh P-selectin determined via filter retention assays (FRA). DNA was radiolabeled, incubated with increasing amounts of proteins and filtrated through a nitrocellulose membrane. Fractions of bound DNAs were detected via autoradiography and quantified. (A) Recombinant human E-selectin incubated with DNA pool after one (▴) and 17 (•) SELEX rounds. (B) Aptamer SDA incubated with rh E-selectin (•, *K*_d_ ≈ 87 nM), rh P-selectin (▪, *K*_d_ ≈ 84 nM), or streptavidin (▴) as a control. A control DNA did neither bind to human E- (▾) nor P-selectin (♦).

With the aim to select DNA aptamers that inhibit the interaction between human E-selectins and their ligands presented on cancer cells, we performed SELEX using recombinantly produced human E-selectin fusion protein as target molecule. After 17 rounds of selection, the enriched DNA pool showed approximately 20% binding to rh E-selectin compared to the initial DNA pool (Figure 3A), comprising a dissociation constant (*K*_d_) of 170±65 nM. Sequence analyses of 50 single oligonucleotides derived from this pool revealed no sequence similarities between these oligonucleotides. However, investigating several of these molecules by filter retention assays, we identified the rh E-selectin specific DNA aptamer SDA (5′-GCCTGTTGTGAGCCTCCTAACGATTTGGATTTGGGGTGGAGGGTATGGTTTGTGCTGGCGTTCTCATTTCCCATGCTTATTCTTGTCTCCC-3′) with a *K*_d_ value of 98±16 nM (Figure 3B).

Due to sequence similarities between E- and P-selectins [6], [22]–[24], we tested the affinity of SDA for recombinant human E-selectin/IgG-Fc-chimeras (rh P-selectin; R&D Systems). Thereby SDA, originally selected for human E-selectin, also showed remarkable affinity for human P-selectin (*K*_d_ = 95±18 nM) but not at all to streptavidin. Finally, control DNA (5'-GCCTGTTGTGAGCCTCCTAACGAGGAGTGGGCTAAAGGTATGTTGTGGGTTTGGTTCCATGCTTATTCTTGTCTCCC-3'), which differed from SDA in the randomized region, did neither bind to rh E- nor rh P-selectin (Figure 3B).

**Figure 4:**
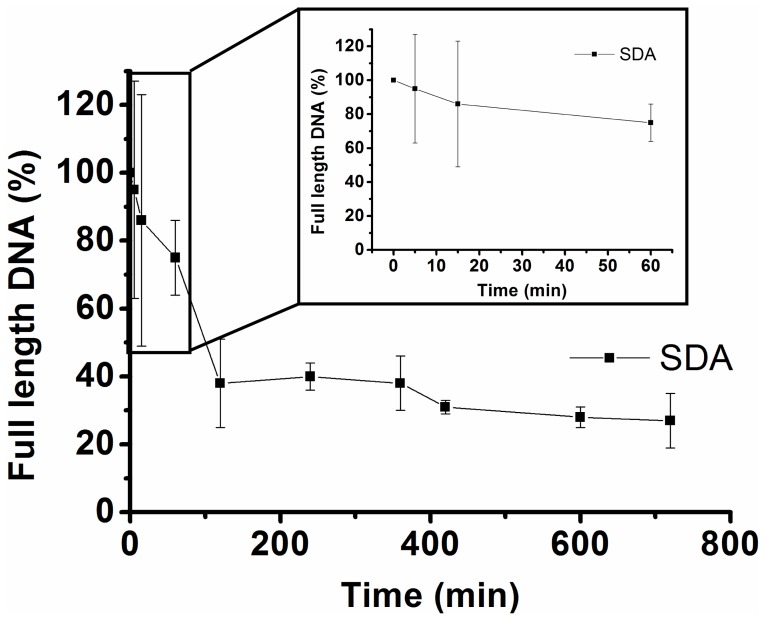
Stability of SDA in cell culture media. Radiolabeled SDA was incubated with full medium and analyzed via polyacrylamide gel electrophoresis. After one hour more than half of SDA was still available (small box).

All attempts to minimize the 91 nucleotide long aptamer failed despite applying Mfold [25], [26] aided secondary structure predictions for predicting shorter sequences. We monitored the stability of SDA in full medium. Even though a slow degradation could be observed, after 12 h more than 25% full length aptamer could still be detected in the absence of selectins (Figure 4).

### DNA Aptamers Inhibit Cell Adhesion to Immobilized Recombinant Human Selectins under Physiological Shear Stress

**Figure 5:**
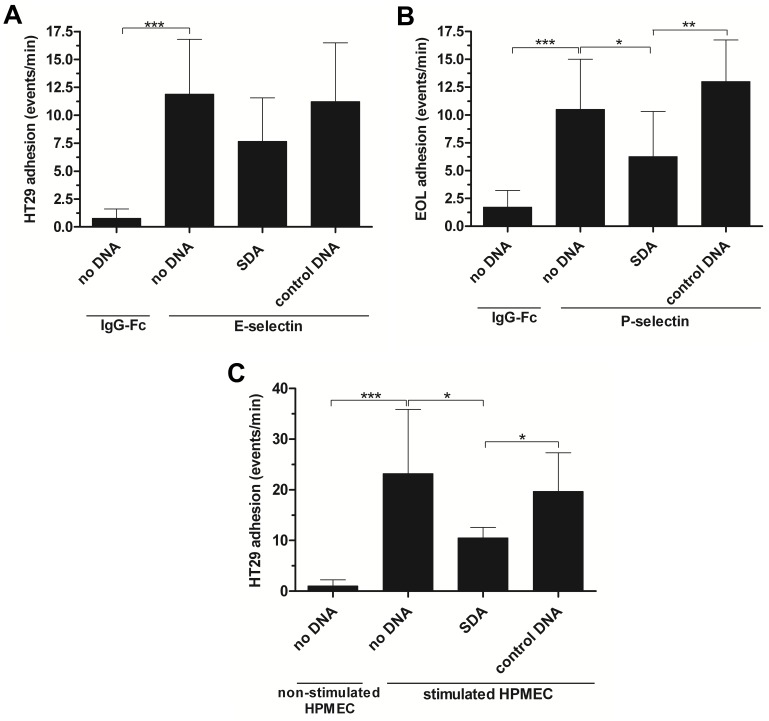
SDA inhibited dynamic cell adhesion. Immobilized rh E- (A) or rh P- (B) selectin (0.2 μM each) as well as stimulated HPMECs (C) were incubated with 5 μM of SDA or control DNA. Selectin ligand-presenting tumor cells were perfused over immobilized proteins or E-selectin presenting HPMECs (flow rate 8 mL/h) and retaining cells were counted. SDA reduced the adhesion to corresponding cells (n = 6 of overall 2 different experiments, *P*-values applied to the selectins). IgG-Fc control showed no adhesion effect. All *P* values were calculated with untreated selectins as standard (* P<0.05, ** P<0.01, *** P<0.001).

We investigated whether the SDA was able to interfere with the adhesion of selectin ligand presenting tumor cells to E- and P-selectins under physiological shear stress conditions. Therefore, rh-selectins were immobilized on a laminar flow micro-chamber and the capillaries were perfused either with HT29 cells in case of human E-selectin or with EOL-1 cells in case of human P-selectin. Adhesive events were recorded. In absence of SDA, HT29 cells adhered on E-selectin-coated chambers with 11.89±4.9 events per minute (Figure 5A). To examine the inhibitory effect of SDA, E-selectin-coated chambers were pre-incubated with SDA. Afterwards, this capillary was perfused with HT29 cells and the number of HT29 adhering cells decreased significantly to 7.67±3.9 events per minute corresponding to a reduction of 35%. When control DNA was used, HT29 cell adhesion was not impaired (11.22±5.3 events/min). As a proper negative control IgG-Fc without any fusion partner was immobilized on a micro-chamber to exclude unspecific binding. This resulted in only rarely binding events (0.78±0.8 event/min, *P*<0.001 *vs.* E-selectin).

To examine if SDA also inhibits EOL-1 cell adhesion to human P-selectin, we immobilized human P-selectin on the chamber’s surface. In the laminar flow assay, unaffected EOL-1 cell adhesion to human P-selectin resulted in 10.42±3.7 events per minute (Figure 5B). This value was reduced to 60% with 6.54±2.2 events per minute in presence of SDA (*P*<0.05 *vs.* P-selectin). Upon incubation with control DNA, flow adhesion of EOL-1 cells to P-selectin remained unchanged with 13.00±3.7 events per minute (*P*<0.01 *vs.* SDA).

### SDA Inhibits HT29 Flow Adhesion to Stimulated Human Pulmonary Microvascular Endothelial Cells

After demonstrating that SDA was able to reduce tumor cell adhesion at human E- and P-selectin-coated surfaces under laminar flow stress, we next investigated the influence of SDA on cell-cell interactions. Therefore, two human cell lines were used: human pulmonary microvascular endothelial cells (HPMECs) and HT29 cells. Non-stimulated HPMECs do not present E-selectin at their surface. Upon TNFα-stimulation, HPMECs produce E-selectin and present it on their cell surface allowing for the interaction with HT29 that carry the E-selectin ligands sLe^X^ and sLe^A^. First, non-stimulated HPMECs were coated on a micro-chamber. Adherence of selectin ligand-presenting HT29 cells was determined to be 1.50±1.3 cells per minute (−rh TNFα). After E-selectin production was induced by treatment with rh TNFα for 4 h prior to the flow adhesion experiments, the number of HT29 cells adhering to HPMECs increased to 23.17±12.7 events per minute (+rh TNFα, *P*<0.01 *vs.* stimulated HPMEC). To investigate the influence of SDA on this cell-cell interaction, rh TNFα-stimulated HPMECs were incubated either with SDA or control DNA. The following laminar flow assay with HT29 cells showed that SDA reduced HT29 adhesion on E-selectin presenting HPMECs significantly to 45% (10.50±2.1 events/min, *P*<0.05 *vs.* stimulated HPMECs). In contrast, control DNA did not show any significant effect (19.67±7.7 events/min, *P*<0.05 *vs. SDA*; Figure 5C).

## Discussion

Metastasis formation is the main clinical hurdle in cancer therapy [2]. A rising number of studies revealed a crucial involvement of E- and P-selectins during the metastatic [4], [27], [28]. Consequently, selectins emerge as promising targets to interfere with metastasis formation. For this purpose, aptamers could be advantageous tools. Aptamers are able to bind their target molecule with high specificity and affinity and can inhibit the function of a target molecule. In contrast to antibodies, these oligonucleotides exhibit temperature stability as well as straightforward and inexpensive synthesis together with uncomplicated handling. Especially DNA aptamers might be more applicable then RNA aptamers regarding their longer half-life in serum. The therapeutic relevance of aptamers has been proven by previous studies [12].

We performed an *in vitro* selection for DNA aptamers binding to E-selectin and identified an aptamer, named *S*electin *D*NA *A*ptamer (SDA) that comprises high affinities for both, human E- as well as for human P-selectin. For SDA a dissociation constant of approximately 100 nM was determined, which documents the high affinity of the bimolecular interaction between the aptamer and the selectin.

Due to amino acid sequence similarities between E- and P-selectin and the same affinity for sLe^X^ and sLe^A^, respectively [6], [22], [23], [29], the comparable affinity of SDA to both selectins is not surprising. *In vitro* binding assays showed an almost similar affinity of SDA for recombinant human P- and E-selectin. Assays with recombinant murine selectins showed that SDA retained affinity for murine selectin as well which was also not unexpected due to the sequence analogy between human and murine selectins (data not shown). We did not prove the possible binding affinity of SDA for L-selectin, because of its lacking importance in the metastasis process. Furthermore L-selectin interacts with other ligands than E- or P-selectin.

As mentioned above, nucleic acids in general and RNA in particular are not remarkably stable in serum due to the presence of various nucleases [12]. To analyze the aptamer's viability, we performed a stability assay with radioactively labeled SDA. The aptamer turned out to be stable to a great extent in full medium for several hours. After one hour about 80% full length SDA could be detected. Furthermore, it is known that aptamers with a mass of approximately 40 kDa or larger remain in circulation for extended periods of time [30]. Thus we would expect a similar behavior for our selectin aptamer with a mass of 30 kDa, which is a requirement for any *in situ* or even *in vivo* applications in coming investigations. This case and the demonstrated stability of the SDA are encouraging features for future successful *in vivo* studies.

As SDA is able to inhibit the adhesion to E- as well as P-selectin, we hypothesized that this aptamer interferes with the lectin domains of the selectins, as those are responsible for the carbohydrate binding [31]. Using dynamic flow adhesion assays, we first demonstrated that SDA inhibited the interaction between E-selectin and selectin ligand presenting HT29 cells as well as the interaction between P-selectin and selectin binding EOL-1 cells in full medium under shear stress conditions. Subsequently, we tested the inhibitory effect of SDA on the interaction of E-selectin presenting HPMECs and selectin binding HT29 cells. This assay simulates the natural adhesion process quite well since it runs under physiological shear stress conditions and we measured a significant reduction for the HT29 adhesion mediated by SDA of 45%. This fact verified the inhibitory efficiency of this aptamer and validated its stability in full medium and subsequently its capability for *in vivo* studies.

To the best of our knowledge, only one thio-modified DNA aptamer (ESTA-1) has been described capable in binding and blocking E-selectin efficiently [32]. This, however, did not show any target binding in our hands in filter retention assays. Furthermore two modified RNA aptamers PF377 [33] and ARC5690 [30] exist that bind and block P-selectin. These or other aptamers with any modification within their sugar moiety are relatively expensive to produce and hence less suitable for long time treatments. In addition, the 2′-fluoro modified RNA aptamer PF377 is limited for any *in vivo* application, since it can only be dealt with synthetic PBS omitting any nucleases [33], [34]. The serum instability of this mentioned and most other RNA aptamers is the main reason for their failure in advanced studies.

Compared to the already existing modified aptamers, we have selected a stable non-modified DNA aptamer with inhibitory capacity on selectin-ligand interactions between vascular endothelium and tumor cells by reducing the initial and rate limiting step during transendothelial migration of cancer cells.

In addition to oligonucleotide antagonists, alternative selectin targeting inhibitors are presented by a substance class named glycomimetics. These are modified oligosaccharides which mimic natural carbohydrates comprising the capability to bind the mimicking carbohydrate ligand. One glycomimetic that blocks E-, P-, and L-selectin is GMI-1070 [9]. The SDA aptamer described here represents a new selectin inhibitor that can be used in combination with already existing other inhibitors to prevent metastasis formation.

## Conclusions

In summary, we have selected a non-modified 91 nucleotide long DNA aptamer, SDA, which shows high affinity for human E- and P-selectin with *K_d_* values of about 100 nM. Its ability to inhibit the interaction of human cancer and leukemia cells to selectins even in a flow chamber simulating the blood flow in physiological environment highlights SDA as a promising tool for further *in* *vivo* studies. The generation of mice harboring corresponding human selectins for those *in* *vivo* assays is one important next step and subject of current experiments.
